# Tafamidis therapy in transthyretin amyloid cardiomyopathy: a narrative review from clinical trials and real-world evidence

**DOI:** 10.1186/s43044-024-00517-y

**Published:** 2024-07-10

**Authors:** Ikponmwosa Jude Ogieuhi, Oshomoh Mark-Anthony Ugiomoh, Kudzaishe Muzofa, Kristen Callender, Johnson David Ayodeji, Nnokam Prayer Nnekachi, Barkavi Thiyagarajan, Emmanuel Obokhai Uduigwome, Abhay Kapoor, Moses Chukwuebuka Odoeke, Reem Gamaleldin Hassan Mohamed, Courage Idahor

**Affiliations:** 1 Siberian State Medical University, Tomsk, Russia; 2 Queen Elizabeth Hospital, Martindales Road, Bridgetown, St. Michael Barbados; 3 V. N Karazin National University, Kharkiv, Ukraine; 4 Ivano Frankivsk National Medical University, Ivano-Frankivsk, Ukraine; 5 Far Eastern Federal University, Vladivostok, Russia; 6 Lagos University Teaching Hospital, Lagos, Nigeria; 7 B. J. Medical College, Ahmedabad, India; 8 Saudi Arabia Ministry of Health, Riyadh, Saudi Arabia; 9 Barking, Havering and Redbridge NHS Trust, London, UK

**Keywords:** Amyloidosis, Cardiomyopathy, ATTR-CM, Tafamidis, Therapeutic intervention, Heart failure

## Abstract

**Background:**

Amyloidosis is a heterogeneous group of disorders caused by the extracellular deposition of insoluble misfolded proteins, leading to end-organ damage. Transthyretin amyloid cardiomyopathy (ATTR-CM) is a subtype in which a protein known as transthyretin accumulates within the heart tissue, progressively resulting in restrictive cardiomyopathy and heart failure. Due to the progressive nature of ATTR-CM, clinical management requires efficacious regimens to manage the debilitating condition and Tafamidis shows promising results in this regard.

**Main body:**

ATTR-CM poses a significant challenge due to its nature and limited therapeutic options. Tafamidis is a novel therapy designed to stabilize the transthyretin tetramers, inhibiting the formation of amyloid fibrils. It has emerged as a promising treatment and the only FDA-approved drug for ATTR-CM. Tafamidis' role in slowing disease progression and improving outcomes in patients with ATTR-CM has been demonstrated in the major randomized control trial ATTR-ACT with promising open-label extension studies, some still ongoing. Additionally, real-world evidence supports its use in clinical practice, showing its role in reducing morbidity and mortality associated with this condition. Clinical evidence shows its efficacy in improving symptoms and cardiac function in patients. Case studies also reveal significant benefits to patients like reducing myocardial damage, reversal of atrial fibrillation, and resolution of heart failure symptoms. Real-world outcomes and clinical trials show a consistent reduction in amyloid deposition, cardiovascular-related hospitalizations, and all-cause mortality with Tafamidis therapy.

**Conclusion:**

Tafamidis is an essential component of the treatment of ATTR-CM and this narrative review synthesizes the current evidence regarding safety, efficacy, and utilization in real practice. While it shows promising effects, its effectiveness may also vary and high cost precludes real-world large-scale studies. Overall, Tafamidis emerges as a valuable therapeutic option for managing ATTR-CM.

## Background

ATTR-CM is a type of systemic amyloidosis characterized by the extracellular deposition of transthyretin (TTR) amyloid fibrils in the myocardium. TTR, also known as pre-albumin, is a 127 amino acid, 55 kDa protein produced primarily in the liver and is a transporter of thyroxine (T4) and the retinol-binding protein-retinol (Vitamin A) complex [1]. In the autosomal dominant hereditary or variant ATTR subtype (ATTRv), the incorporation of pathogenic variant subunits into TTR heterotetramers leads to TTR tetramer destabilization, whereas in the wild-type ATTR subtype (ATTRwt) dissociation, misfolding, and aggregation of tetramer are caused by age and not a mutation. This latter type was previously called senile systemic amyloidosis [1, 2].

There are more than 120 pathogenic mutations in TTR resulting in variable phenotypic presentations [3], but the most common mutation (Val122Ile) occurs in approximately 3.4% of the African American population and exclusively has cardiac involvement [4]. The ATTR-ACT trial demonstrated a high prevalence of these three TTR mutations: Val122Ile, Thr60Ala, and Ile68Leu [5].

The accumulation of TTR amyloid fibrils in the cardiac muscle results in ventricular wall thickening and reduced elasticity. This manifests as a diastolic dysfunction and progresses into restrictive cardiomyopathy [5]. Infiltration of the conduction system can lead to conduction disease and atrial arrhythmias. On histopathology, these deposits appear with a green birefringence when viewed under cross-polarized light after staining with Congo red [6].

ATTR-CM is an under-identified etiology of heart failure. While echocardiography and cardiac magnetic resonance are commonly used diagnostic modalities, emerging data highlights the potential of Technetium-99m-labeled 3,3-diphosphono-1,2-propanodicarboxylic acid (99m Tc-DPD) scintigraphy [3, 7, 8]. This non-biopsy method is both sensitive and specific and can detect subclinical amyloid deposition before the onset of heart failure symptoms, an increase in left ventricular mass, or a rise in cardiac biomarkers [9]. Up to 15% of older adults with heart failure may have unrecognized ATTRwt as suggested by recent studies [3]. Early diagnosis is an important determinant of ATTR-CM prognosis, with genetic counseling and familial screening in cases of ATTRv [10, 11].

Early diagnosis and treatment of ATTR-CM results in improved outcomes. Tafamidis is the only FDA-approved therapy for the treatment of ATTR-CM. A study evaluating the efficacy of Tafamidis in subjects with familial amyloid polyneuropathy showed the potential of Tafamidis to decrease neurological progression but did not lead to a statistically significant result and hence was not approved by the FDA [12].

Tafamidis, a non-NSAID benzoxazole derivative, is a monovalent TTR kinetic stabilizer that inhibits the crucial rate-limiting step of TTR amyloidogenesis via selective binding with negative cooperativity (KdS ~ 2 nM and ~ 200 nM) to one of two normally vacant thyroxine-binding sites of the native tetramer. Single-site binding corresponds with complete stabilization [13] of the weaker dimer–dimer interface against dissociation under both denaturing and physiological conditions [14].

The rate-limiting step involves the dissociation of the native TTR tetramer into monomers. Monomers lose their original form in a process known as misfolding. They cluster to form oligomers which aggregate to form insoluble amyloid fibrils. Tafamidis acts on the crucial rate-limiting step by inhibiting dissociation, hence stabilizing the tetramer and reducing the availability of amyloid fibrils.

Tafamidis resulted in reduced cardiovascular-related events and all-cause mortality in the ATTR-ACT trial [15, 16]. Tafamidis improved the survivability of patients with NYHA Classes I–III, but the most effect was seen in patients with Classes I and II. While there were reduced cardiovascular-related hospitalizations in NYHA I and II, there was a paradoxical increase in those who had NYHA III symptoms [16]. The increased rate of hospitalization in this subgroup is likely due to the increased survival in this subgroup of patients with advanced severity of disease [17]. However, further analysis up to 58 months of follow-up revealed statistically divergent curves of survival benefit in the NYHA Class III subset that had little benefit in the original trial [18].

## Methodology

A literature search was conducted to identify relevant studies and publications on Tafamidis' efficacy in managing ATTR-CM. Table 1. Databases such as PubMed, MEDLINE, EMBASE, and Cochrane Library were searched. The search strategy included keywords such as "Tafamidis," "transthyretin cardiac amyloidosis," "cardiac disease," "Tafamidis efficacy," "clinical trials," "cardiac disease treatment," and related terms. Inclusion criteria include studies reporting on the use of Tafamidis in managing ATTR-CM, including clinical trials and observational studies. Non-English language studies were excluded. No time limit was placed on the search. Two reviewers conducted the selection process independently, with disagreements resolved through discussion or consultation with a third reviewer. A qualitative narrative analysis approach was employed to explore and synthesize the narrative elements present in the selected studies. This involved identifying common themes and patterns.

**Table 1:** Studies from clinical trials

Author and year	Study design Duration	Population/sample size	Intervention	Outcomes	Adverse events
*Parent Clinical Trial*					
Maurer et al. [5] NCT01994889	Phase III, double-blind, placebo-controlled, multicenter, randomized 2:1:2 Duration: 30 months	*n*: 441 ATTRv-CM (24%)	Oral Tafamidis 80 mg daily, Tafamidis 20 mg daily, or placebo	*Primary outcomes:* Lower all-cause mortality (78 of 264 [29.5%] vs. 76 of 177 [42.9%]; hazard ratio, 0.70; 95% confidence interval [CI] 0.51–0.96) *Secondary Outcomes:* Lower rate of cardiovascular-related hospitalizations, with a relative risk ratio of 0.68 (0.48 per year vs. 0.70 per year; 95% CI, 0.56 to 0.81) A lower rate of decline for the 6MWT and the KCCQ-CSS (*p* < 0.001 for each)	Dose reduction related to adverse events: 0.8% for Tafamidis vs. 2.3% for placebo Treatment-related TEAEs were reported by 44.9%, 38.6%, 50.8% of patients in the Tafamidis 80 mg, 20 mg, and placebo groups
*Long-term extension (LTE) study*					
Elliot et al. [18] NCT02791230	LTE of ATTR-CT Open Label Study Assessing long-term efficacy of Tafamidis Duration: Median 58.5 months (continuous Tafamidis) and 57.1 months (placebo-Tafamidis)	353 Continuous Tafamidis (*n*:176) Placebo to Tafamidis (*n*:177)	ATTR-ACT Tafamidis subgroup continued on the same dose (80 mg or 20 mg) ATTR-ACT placebo group randomized in 2:1 ratio to Tafamidis 80 mg or 20 mg doses Protocol amendment caused switch to 61 mg dose	Significant mortality reduction in ATTRv (*p* = 0.05), TTR-wt (*p* = 0.006), NYHA Class I & II (*p* = 0.003), and NYHA Class III (*p* = 0.06) Comparable safety profile between Tafamidis 80 mg and 20 mg	No dose reductions related to adverse events Death: 44.9% in continuous Tafamidis group vs. 62.7% in placebo-to-Tafamidis group (hazard ratio: 0.59 [95% CI: 0.44–0.79]; *p* < 0.001)
*Post hoc Analysis*					
Shah et al. [23]	Exploratory post hoc analysis of ATTR-CT patients randomized to Tafamidis 80 mg daily compared to placebo Duration: 30 months	*n*: 176 (Tafamidis 80 group) *n*: 177 in placebo group	See ATTR-CT [10]	Attenuated decline in these parameters at 30 months LVSV 7.02 mL (95% CI, 2.55–11.49; *p* = .002) LV GLS − 1.02% (95% CI, − 1.73 to − 0.31; *P* = .005) Septal *E*/*e* − 3.11 (95% CI, − 5.50 to − 0.72; *p* = .01) Lateral *E*/*e*′. − 2.35 (95% CI, − 4.01 to − 0.69; *P* = .006) Attenuated decline in regional measures of strain	N/A
Hanna et al. [24]	Post hoc analysis assessing Tafamidis’ efficacy in health-related QOL	441	ATTR-CT [10] Pooled Tafamidis (20 mg and 80 mg) groups and placebo	Patient global assessment (PGA) improvement with Tafamidis vs placebo (42.3% vs. 23.8%) KCCQ-OS score improvement with Tafamidis vs. placebo (41.8% vs. 21.4%)	N/A
Garcia-Pavia et al. [25]	Post hoc analysis of ATTR-CT and its LTE study assessing Tafamidis 80 mg dose in octogenarians Duration: 30 months (ATTR-CT) Duration: An additional 60 months (LTE) NCT02791230	< 80 age group (*n*: 265) Tafamidis 80 125 Placebo 140 > 80 age group *(n:* 88*)* *n*: 51 (Tafamidis 80) *n*:37 (Placebo)	See ATTR-CT [10] LTE [1]—All groups including placebo group received Tafamidis	Significant least squares mean difference from baseline in 6MWT, KCCQ-OS score, NT-proBNP at 30 months in age > / = 80 Longer median survival for ages < / = 80 continuous Tafamidis treatment in the LTE (80 vs 41 months; HR = 0.4513 [95% CI: 0.3176–0.6413]; P < 0.0001) than those initially on placebo	N/A
Rozenbaum et al. [19]	Flexible probabilistic multi-state, cohort, Markov model	264	(1) 30-month follow-up data from ATTR-ACT assessing SoC (*n* = 177) vs. the pooled Tafamidis cohorts (20 mg dose and 80 mg dose; *n* = 264) and (2) 49-month follow-up data from the open-label LTE for continued Tafamidis treatment since the start of ATTR-ACT (*n* = 264)	Predicted mean survival 6.73 years for Tafamidis and 2.85 years for the standard of care (SoC), Incremental mean survival of 3.88 years [95% confidence interval (CI) 1.32–5.66] with Tafamidis	N/A
Sperry et al. [26]	Post hoc analysis of ATTR-CT assessing the efficacy of Tafamidis in NYHA Class III subset	441	Tafamidis meglumine, 80 mg or 20 mg (pooled cohort), vs. placebo	For NYHA Class III subset (vs. placebo): Subjective improvement in health status at 30 months (35% vs 10%) Subjective decline in health status at 30 months	
Damy et al. [11]	Post hoc analysis of ATTR-CT and LTE assessing efficacy and safety of different Tafamidis doses	441	See ATTR-CT	Significant reduction in combined all-cause mortality and CV-related hospitalizations by both 80 mg (*p* = 0.0030) and 20 mg (*p* = 0.0048) Greater survival benefit with 80 mg dose vs 20 mg dose [0.700 (0.501–0.979), *p* = 0.0374] Comparable safety endpoints in LTE for both doses	Death (80 mg, 20 mg, placebo): 27.8%,26.1%, 40.7%) UTI was the most common TEAE in 20 mg dose (5.7%) vs 2.3% and 4.5% in 80 mg and placebo, respectively Diarrhea was the most common TEAE in 80 mg dose (8%) vs. 2.3% and 10.2% in the 20 mg dose and placebo, respectively
Rapezzi et al. [10]	Post hoc analysis of ATTR-ACT assessing efficacy of Tafamidis in ATTR subtypes	441 ATTRwt (n: 335) ATTRv (n: 106)	See ATTR-CT	No significant reduction in mortality between ATTRwt (*p* = 0.0875) and ATTRv (*p* = 0.1667) Both subtypes had similar reduction in the 6MWT and KCCQ-OS score with Tafamidis treatment compared to placebo 6MWT: ATTRwt (77.14 ± 10.78; *p* < 0.0001) ATTRv (79.61 ± 29.83 m; *p* = 0.008) KCCQ-OS score: ATTRwt (12.72 ± 2.10; *p* < 0.0001) ATTRv (18.18 ± 7.75; *p* = 0.019)	N/A
Elliot et al. [16]	Interim Analysis of LTE Post hoc analysis of ATTR-CT Assessing impact of Tafamidis 80 on survival in NYHA Class III subset Duration: Up to median 56 months	192	All patients in the LTE transitioned to Tafamidis free acid 61 mg (bioequivalent to the FDA-approved 80 mg dose) following a protocol amendment	Reduced risk of all-cause mortality across NYHA Classes I/II/III favoring continuous Tafamidis vs initial placebo All-cause mortality (continuous Tafamidis vs placebo-to-Tafamidis): NYHA Class III: 64% vs 81% NYHA Class I/II: 41% vs. 61%	N/A

## Main text

### Rationale for an in-depth analysis of clinical trials and real-world evidence

Further study and analysis of the use of Tafamidis in the management of ATTR-CM is pivotal as there is still a lot to learn. With the median overall survival on Tafamidis not attained since its approval in 2019, the full potential of this disease-modifying therapy is yet to be discovered [19]. While the impact of Tafamidis on all-cause mortality and cardiovascular-related hospitalization is remarkable little is known about the autonomic effect, post-therapeutic quality of life, and other cardiovascular parameters, and lack of evidence of the degree of stabilization to which approved doses exhibit fullest potential [13]. Most real-world studies lack large sample sets, sufficient follow-up periods, and equal representation of the subtypes precluding the availability of evidence regarding long-term efficacy and generalizability associated with Tafamidis treatment [20]. A noteworthy lack of real-world studies evaluating changes in a comprehensive set of echocardiographic parameters after Tafamidis treatment at follow-up exists in the literature. Subsets like advanced age and patients in more advanced stages of disease can benefit from longer follow-up analysis. Despite the burden of ATTR-CM on black populations, there remains a paucity of studies focusing on the efficacy and safety of this drug in that subset. Notably, more than 30% of the world [21] are without access to this costly drug creating a void in knowledge on the impact of Tafamidis on these diverse populations. This geographical disparity stems from a lack of available clinical registries/cohorts on ATTR-CM in countries outside of Europe and North America [22].

### Mechanism of action of Tafamidis

**Fig. 1:**
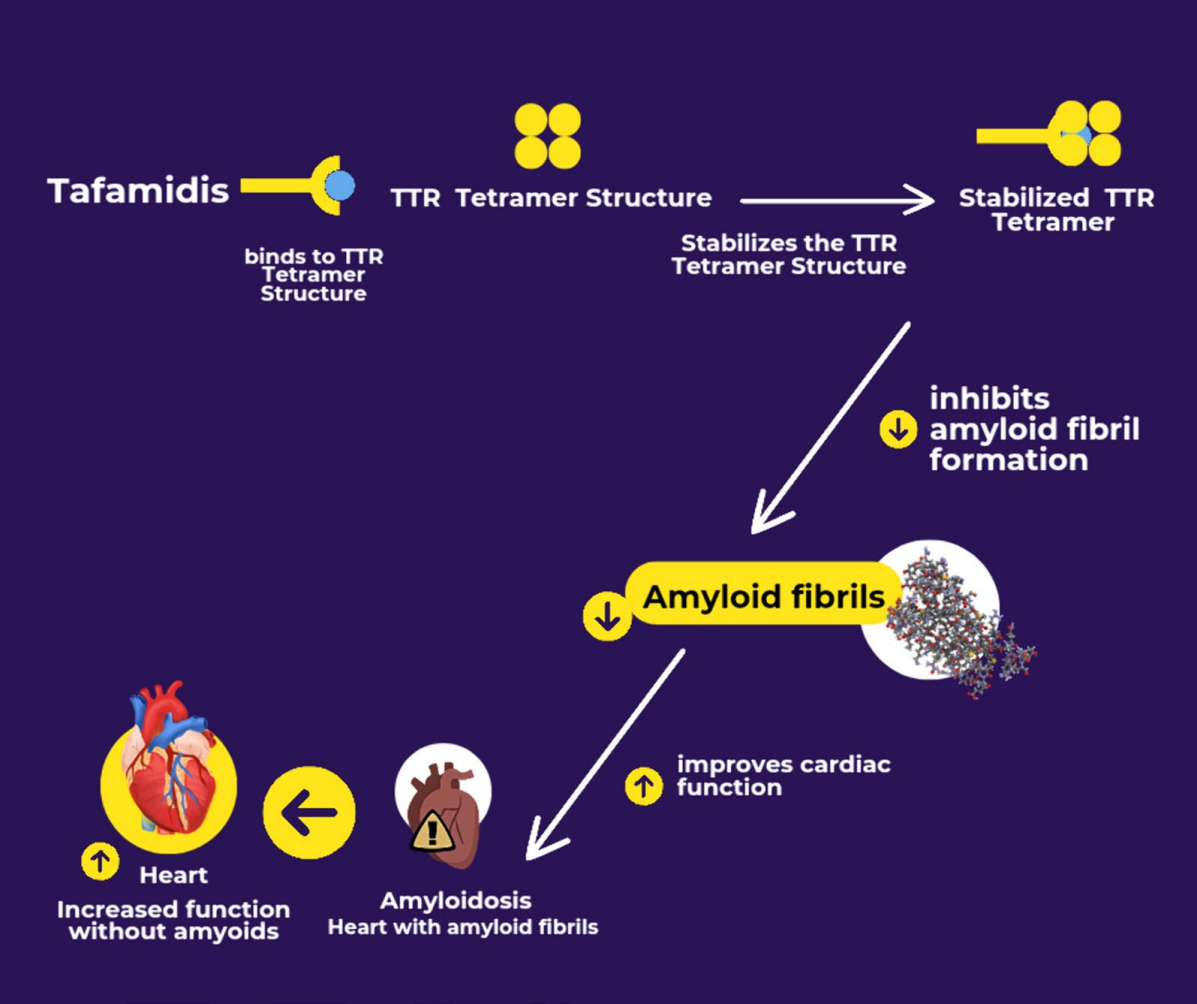
Mechanism of Action of Tafamidis

Tafamidis is a small molecule that stabilizes the transthyretin (TTR) protein, preventing its dissociation into monomers, which can misfold and form amyloid fibrils. In the context of cardiac amyloidosis, Tafamidis binds selectively to the thyroxine-binding sites of TTR tetramers, enhancing their stability. This binding inhibits the dissociation of TTR into monomers, which is a critical step in the amyloidogenic cascade, leading to amyloid fibril formation and subsequent deposition in cardiac tissue [1] (Fig. 1).

In hereditary transthyretin amyloidosis (ATTR), mutations in the TTR gene result in a variant TTR protein that is more prone to misfolding and aggregation. Tafamidis effectively stabilizes both wild-type and variant TTR tetramers, reducing the formation of toxic amyloid aggregates. By stabilizing TTR, Tafamidis reduces amyloid deposition in the heart, which can improve cardiac function and patient outcomes. Clinical studies have shown that Tafamidis treatment slows the progression of cardiomyopathy in patients with ATTR amyloidosis, highlighting its therapeutic potential [10, 15].

## Overview of clinical trials (Table 1)

### Efficacy of Tafamidis in clinical trials

In this review article, we aim to summarize the major clinical trials that investigate Tafamidis as well as pay close attention to the study designs, population, endpoints, and evolution of trial methodologies over time to get a more nuanced and deep understanding of the mechanics of this drug [23].

ATTR-ACT is a multicenter, placebo-controlled, double-blinded, randomized control trial that investigated the safety and efficacy of Tafamidis in ATTR-CM. Endpoints included all-cause mortality, cardiovascular-related hospitalizations, cardiac function, functional status, and quality of life from baseline to 30 months. Health and functional status were measured using the KCCQ-OS score and 6MWT, respectively. Tafamidis was associated with a reduction in all-cause mortality cardiovascular-related hospitalizations and functional decline compared to placebo. Significant changes from baseline to month 30 in LVEF (*p* = 0.13), LV GLS (*p* = 0.005), Septal *E*/*e*′ (*p* = 0.01), and Lateral *E*/*e*′ (*p* = 0.006) were observed with Tafamidis treatment. There was also a comparable safety profile to placebo [5].

An LTE study sought to determine the long-term efficacy and safety profiles of Tafamidis in ATTR-CM. Hence, endpoints from the parent trial were monitored over a prolonged follow-up period of a median of 58.5 months in the continuous Tafamidis group and 57.1 months in the placebo-to-Tafamidis group. Unlike the parent trial, there were no dose reductions related to adverse events. Continuous Tafamidis treatment was associated with a lower rate of death than in the placebo-to-Tafamidis group [10].

Damy et al. [11] investigated the efficacy and safety of different doses of Tafamidis in the ATTR-ACT trial and its LTE. Even though both doses had comparable safety and efficacy endpoints, greater survival benefit was shown with the 80 mg dose.

Rapezzi et al. evaluated the efficacy of Tafamidis in patients with both wild-type ATTR-CM (ATTRwt-CM) and hereditary ATTR-CM (ATTRv-CM) and found comparable reduction in 6MWT, KCCQ-OS scores and mortality reduction between the subgroups [10].

Hanna et al.'s [24] study investigated Tafamidis' efficacy in health-related quality of life and concluded that Tafamidis significantly reduced the decline in all 4 KCCQ-OS domains (*p* < 0.0001 for each). A larger proportion of Tafamidis-treated reported their health status as "improved" at 6-month checkpoints until month 30.

Shah et al.'s [23] pre-specified analysis focused on the Tafamidis (80 mg dose) impact on echocardiographic parameters and regional strain patterns that were not fully explored in prior studies of other ATTR-CM therapeutics. The onset of decline in LVEF is earlier in placebo (6 months) than in the Tafamidis 80 mg group which only became apparent at 30 months.

Garcia-Pavia et al. [25] investigated the efficacy of Tafamidis among octogenarian patients with ATTR-CM using data from the ATTR-ACT trial and ongoing LTE studies. Efficacy was comparable in both age groups with Tafamidis treatment. There was a significant trend in increased median survival in those < 80 on continuous Tafamidis compared to those who were initially in the placebo treatment group. In contrast, continuous Tafamidis had no significant increase in survival in the > 80 subgroups in the LTE.

Two studies analyses extracted and evaluated data from the NYHA Class III subset of ATTR-ACT [26, 27]. Sperry et al. found that Tafamidis treatment resulted in better health status compared to placebo in patients with NYHA Class III symptoms. Tafamidis was associated with stability and improved health status at 30 months, regardless of NYHA Class [26].

An extrapolation model revealed that patients spend more years in lower NYHA Classes than standard care, suggesting its crucial role in slowing disease progression. From a NYHA Class I/II baseline, the projected increase in life years and quality-adjusted life years (QALYs) is 5.49 and 3.29 to 4.62, respectively, with Tafamidis compared to the standard of care (SoC). Even at a baseline of NYHA Class III, Tafamidis treatment projected a 27.4% improvement compared to the standard of care [26].

## Safety profile from clinical trials

In the ATTR-ACT trial, the proportion of treatment-related treatment-emergent adverse events (TEAEs) reported by the Tafamidis 80 mg, 20 mg, and placebo groups were (44.9%), (38.6%), and (50.8%), respectively. A greater proportion of deaths was observed in the placebo group (40.7%) and the majority of deaths in the Tafamidis groups were attributed to the result of disease severity. Cardiac failure was the most common cause of TEAEs in all groups. The most common TEAE associated with Tafamidis 80 mg dose was diarrhea (18%) and urinary tract infection with Tafamidis 20 mg dose (5.7%). Two patients in the Tafamidis group requested a dose reduction compared to four patients in the placebo group [5]. Unlike the ATTR-ACT trial, no TEAE-related dose reductions were recorded. A higher proportion of death was observed in the placebo-to-Tafamidis group compared to the continuous treatment group. (62.7% vs. 44.9%) Comparable incidences of severe adverse events were noted between 80 and 20 mg doses [5]. Overall, safety and tolerability were comparable in both doses and placebo.

It should be noted that the majority of the case reports and clinical trials used earlier in this section were funded by the drug manufacturer. Independent case reports show a slight deviation from the results obtained by case reports obtained by manufacturer-backed reports.

## Comparative analysis (Table 2)

**Table 2:** Comparative analysis and real-world evidence

Drug	Study name (year), NCT number, status	Study design Duration	Population and intervention	Outcomes
*TTR kinetic stabilizers (monovalent)*				
Tafamidis	ATTR-ACT [24] NCT01994889 Completed	Phase III, multicenter, double-blinded, placebo-controlled, Randomized, 2:1:2 Duration: 30 months	*n*: 441 ATTR-CMv (24%) and ATTRwt-CM Oral Tafamidis 80 mg daily, Tafamidis 20 mg daily (*n*:264) or placebo (*n*:177)	*Primary outcome* All-cause death 29.5% of Tafamidis group vs. 42.9% of placebo group (*p* < 0.05) *Secondary Outcomes* Cardiovascular-related hospitalization: 0.48 per year for Tafamidis vs. 0.70 per year for placebo (*p* < 0.05) Tafamidis was associated with a lower rate of decline for the 6MWT and the KCCQ-CSS (p < 0.001 for each) *Adverse events (Tafamidis vs placebo)* Similar safety profile Dose reduction related to adverse events in 0.8% for Tafamidis vs. 2.3% for placebo
Acoramidis	ATTRIbute-CM [27] NCT03860935 Completed	Phase III, double-blinded, placebo-controlled, randomized 2:1 Duration: 30 months	*n*: 632 ATTR-CMv and ATTRwt-CM (90.4%) Oral Acoramidis 800 mg twice daily (*n*: 421) or placebo (*n*:211) Concomitant Tafamidis use: 18%	*Primary outcome* A win ratio of 1.8, 95% confidence interval [CI] 1.4–2.2, *p* < 0.0001) in favor of Acoramidis over placebo for the hierarchal composite of all-cause mortality, cardiovascular-related hospitalization, change from baseline NT-proBNP, and change from baseline in 6MWT *Secondary Outcomes* All-cause mortality: 19.3% vs. 25.7%; hazard ratio (HR) 0.77, 95% CI 0.54–1.10 (*p* = 0.15) Adjusted mean factor change in NT-proBNP from baseline: 0.529 (95% CI 0.46–0.60, *p* < 0.05) Improvement from baseline in 6-min walk distance: 39.6 m (95% CI 21.1–58.2, *p* < 0.001) CV-related hospitalization: 26.7% vs. 42.6% (p < 0.0001) Least means square change in KCCQ-OS: 9.94 points (95% CI 5.97–13.91, *p* < 0.001) *Adverse events (Acoramidis vs. placebo)* 98.1% and 97.6%, respectively *Serious adverse events* 54.6% and 64.9%
*Anti-sense oligonucleotides*				
Eplontersen	CARDIOTTRansform NCT04136171 Active, not recruiting Estimated completion in 2025	Phase III, placebo-controlled double-blinded, randomized Duration: 140 weeks	*n*: 1436 Eplontersen and matching placebo subcutaneous injection every 4 weeks	*Primary outcome *(Baseline to Week 140) Composite Outcome of Cardiovascular (CV) Mortality and Recurrent CV Clinical Events *Secondary Outcomes *(Baseline to Week 121) Change from baseline in the 6MWT Change from baseline in the KCCQ-SS CV clinical events up to week 140 CV mortality up to week 140 All-Cause mortality up to week 140
Inotersen	NEURO-TTR (2018) [35] NCT01737398 Completed	Phase III, double-blinded, placebo-controlled, randomized 2:1 Duration: 65 weeks	*n*: 173 hATTR-PN with or without CM CM subset (*n*: 108, 63%) based on > 1.3 cm interventricular septum thickness on Echo at baseline 300 mg of Inotersen (*n*:75) and matching placebo (*n*:60) subcutaneous injection three times on alternate days in first week followed by once weekly for 64 weeks	*Secondary Outcome (CV-related):* No significant differences in global longitudinal strain and other Echo variables between Inotersen and placebo group Inotersen was associated with a significant improvement in physical component summary score of SF-36 Overall, Inotersen improves QOL in patients with hATTR-PN who have greatest disease burden, i.e., those with CM *Adverse events* 78% in Inotersen treatment group vs. 38% in placebo *Serious adverse events* Total number of deaths of any cause: 5 (all in Inotersen intervention arm) Thrombocytopenia associated with intracranial hemorrhage (*n*:1) Glomerulonephritis (*n*: 3, 3% Inotersen intervention arm)
*Small Interfering RNA*				
Patisiran	APOLLO-B (2022) [37] NCT03997383 Completed	Phase III, placebo-controlled, double-blinded, randomize 1:1 Duration: 12 months	*n*: 359 ATT-CMv and ATTwt-CM (81%) Patisiran given at 0.3 mg/kg IV (max dose: 30 mg) every 3 weeks for 12 months or placebo Concomitant Tafamidis use: 25%	*Primary outcome* Change at 12 months compared with baseline in 6MWT for Patisiran vs. placebo, was: − 8.5 vs. − 21.4 m; difference: 14.69 (*p* = 0.02) *Secondary Outcome* Change in KCCQ-OS from baseline: − 3.4 vs. 0.3, difference 3.7 (*p* = 0.04) Change in NT-proBNP from baseline: 131 vs. 518 (*p* < 0.05) All-cause mortality/cardiovascular events/change in 6MWD: win ratio 1.27 (95% confidence interval 0.99–1.61) All-cause mortality (safety analysis): 3% vs. 4% *Adverse events* Infusion-related reactions Vitamin A deficiency
Revusiran	ENDEAVOUR (2020) [41] Terminated	Phase III, multicenter, placebo-controlled, double-blinded, randomized 2:1	*n*: 206 ATTRv-CM Revusiran (*n*:140) 500 mg subcutaneous daily for 5 days then weekly for 18 months or placebo (*n*:66)	*Severe adverse events* 12.9% death rate with Revusiran compared to placebo (3.0%)
Vutrisiran	HELIOS-B NCT04153149 [39] Active	Phase III, placebo-controlled, randomized, double-blinded Duration: 30–36 months	*n*: 655 Vutrisiran 25 mg subcutaneously once every 3 months during the double-blind period or placebo	*Primary Outcomes* Composite Endpoint of All-Cause Mortality and Recurrent Cardiovascular (CV) Events *Secondary Outcomes* Change from Baseline in 6-minute walk test (6MWT) at Month 30 Change from Baseline in the KCCQ-OS at Month 30 Change from Baseline in Mean Left Ventricular (LV) Wall Thickness by Echocardiographic Assessment at Month 30 Change from Baseline in Global Longitudinal Strain by Echocardiographic Assessment at Month 30 Composite Endpoint of All-Cause Mortality and Recurrent All-cause Hospitalizations and Urgent HF Visits All-cause Mortality Rate of Recurrent CV Events (CV Hospitalizations and Urgent HF Visits) Change from Baseline in NT-proBNP at month 30

### TTR kinetic stabilizers (monovalent)

1. Acoramidis: Acoramidis is also a TTR stabilizer with proven superiority to placebo for improving both cardiovascular and quality of life endpoints with a remarkable 90% TTR reduction [27]. On February 5th, 2024, the FDA announced its acceptance of its new drug application (NDA) based on the positive results from the ATTRIbute-CM trial.
2. Diflunisal: Diflunisal is a nonsteroidal agent which also acts on the rate-limiting step of the amyloidogenesis cascade. Its advantageous structural similarity to thyroxine (T4) allows it to bind to the vacant TTR binding site. Its use in ATTR-CM has been limited to single-arm studies which supported tolerability and safety of the drug [28, 29]; however, it is not FDA-approved for the treatment of ATTR-CM but has off-label use in ATTR-CM and Hereditary Transthyretin Amyloidosis with Polyneuropathy with or without cardiomyopathy. Adverse events include typical nonsteroidal anti-inflammatory drug (NSAID) side effects: bleeding, hypertension, and renal dysfunction. It is more cost-effective and primarily used in the pre-Tafamidis era and in those patients who cannot afford Tafamidis [30].
3. Tolcapone: Tolcapone, a catechol-O-methyltransferase inhibitor, is another monovalent kinetic stabilizer modeled from the "super stabilizer" Mds84. It is a stronger aggregator inhibitor than Tafamidis and binds with the highest affinity to wtATTR than other stabilizers [31]. Mds84, a bivalent TTR ligand that has the advantageous binding to both unoccupied thyroxine-binding sites of TTR, has not been evaluated in clinical trials. An alternative pathway involving proteolysis-mediated TTR fibrillogenesis in vivo that can only be inhibited by simultaneous binding of both sites has been proposed [32].

### Anti-sense oligonucleotides

1. Inotersen: Inotersen, an antisense oligonucleotide, affects the early stages of amyloidogenesis via TTR gene silencing with a weak reduction in TTR concentration (68%) after 15 months of treatment [33–35]. The NEURO-TTR trial evaluated the efficacy and safety of Inotersen in patients primarily with hATTR-PN with a subgroup analysis of the cardiomyopathy (CM) subset. This trial lacked sufficient power to evaluate efficacy in the CM subset for two reasons: 1. Patients assigned to receive Inotersen had more advanced neuropathy who likely had concomitant cardiomyopathy meant that a higher proportion of patients with cardiomyopathy received Inotersen 2. Exclusion criteria saw more end-stage disease being excluded and the likelihood that these patients had CM meant that the majority of the CM subset were not included in the trial [35].

### Small interfering RNA

1. Patisiran: Patisiran, a small interfering RNA agent, is FDA-approved for the treatment of polyneuropathy in patients with hATTR however the FDA rejected its use for ATTR-CM stating "insufficient evidence of clinical meaningfulness" [36]. It is approved for continued use in the ongoing APOLLO-B open-label LTE. When administered intravenously it is superior to placebo for improvement of QOL endpoints via 80% TTR knockdown compared to Tafamidis' 92% reduction [37]. Patisiran significantly improved QOL assessed by KCCQ-OS, 6MWT at 12 months. Of note, researchers did not observe any benefit of Patisiran in those patients also on Tafamidis (25% of the cohort). Studies have supported Patisiran's superiority over Tafamidis regarding treatment effects in patients with hATTR-PN but no comparative study for ATTR-CM between these two exists [38].
2. Vutrisiran: Another promising small interfering RNA, Vutrisiran which is now being evaluated for its clinical efficacy and safety in ATTR-CM in the HELIOS-B Trial, has a potential for "99% TTR knockdown with once annual dosing." Vutrisiran was approved in June 2022 for the treatment of hATTR-PN [39]. Vutrisiran significantly improved neuropathy scores and QOL at nine months in patients with hATTR-PN and had an acceptable safety profile [40].
3. Resuviran: In the ENDEAVOUR trial, evaluating another small interfering RNA, Resuviran was terminated due to high treatment-related mortality compared to placebo [41].

Both Patisiran and Tafamidis have acceptable safety profiles and no monitoring is required during studies [42]. On the other hand, treatment with Diflunisal and Inotersen warrants close monitoring of platelet count and renal function prior to and throughout treatment.

In the first comparison of efficacy and safety between Tafamidis and Diflunisal, there was no significant difference in the progression of biomarkers (NYHA *p*:0412, BNP *p*:0.890, Troponin: 0.015) at 1 year between groups. Diflunisal was associated with a significantly higher transthyretin trend and greater reduction in estimated glomerular filtration rate (EGFR) (*p*: 0.009, 0.008, respectively) [43].

Overall, a paucity of head-to-head studies comparing the efficacy and safety of these novel therapies precludes an effective comparison.

## Real-world evidence (Table 2)

### Real-world data on the outcomes and efficacy of Tafamidis outside of clinical trials for ATTR-CM is lacking

A meta-analysis of 15 studies comprising 2765 patients revealed a significant Tafamidis-associated reduction in the composite endpoint of all-cause death, heart transplant, heart assist device implantation, heart failure exacerbations, and hospitalization in both ATTR types [20].

A predominance of the ATTRwt vs. ATTRv subtype (1060 vs. 78) is noteworthy and underscores unequal representation in studies. Findings support a significant decrease in LVEF (SMD: − 0.17; 95% confidence interval (CI) − 0.31 to − 0.03; *p* = 0.02) but no significant difference in interventricular septal thickness or global longitudinal strain after Tafamidis treatment over a mean follow-up duration of 18.7 ± 17.1 months. A significant decrease in the primary endpoint of all-cause mortality and heart transplantation was associated with Tafamidis treatment compared to placebo (the pooled RR 0.44; 95% CI 0.31–0.65; *p* < 0.01) although no significant difference in primary endpoint occurred between wtATTR or hATTr. (RR 0.44; 95% CI 0.27–0.73 vs. 0.21; 95% CI 0.11–0.40, *p* = 0.08; I 2 = 68%).

These studies all agreed with the study by Maurer et al. [5] which demonstrated a smaller decline in LVEF with Tafamidis treatment compared to placebo.

Real-world studies evaluating changes in myocardial function and echocardiographic features at follow-up after Tafamidis treatment are also lacking.

Ichikawa et al. [44] analyzed changes in LVEF, left ventricular mass index (LVMI), the ratio of peak early diastolic mitral flow velocity, and pulsed-wave Doppler-derived early diastolic velocity from the septal mitral annulus (*E*/*e*′), LA volume index, GLS, and relative apical sparing from baseline in 41 patients with biopsy-proven ATTR-CM before and mean 16 = /–8 months after Tafamidis treatment with standard speckle-tracking. Subgroup comparisons of NYHA class and age (> or < 80) were also conducted. Again, a predominance of ATTRwt-CM subtype existed representing 82.9% of the sample set compared to 17.1% for ATTRv-CM. Even though there was no significant change in parameters after Tafamidis treatment, a reduction of worsening in aforementioned echocardiographic features after Tafamidis in the elderly and those with advanced disease was remarkable [44].

In disagreement, another retrospective study investigating Tafamidis-associated echocardiographic details showed less deterioration of GLS in treated vs untreated patients over 1 year [1.1% IQR 0.95 vs. 0.3% IQR1 *p*:0.02) [45]. This study reported lesser deterioration of myocardial work index and myocardial efficiency in the Tafamidis cohort. This study was also limited by its small sample size and under-represented hATTR subtype. As these patients were treated with Tafamidis meglumine 61 mg, these findings cannot be extrapolated to represent alternative non-bioequivalent dosing.

A much larger retrospective observational cohort study enrolled 842 patients and evaluated the effect of Tafamidis on primary endpoints of the rate of heart failure exacerbations and all-cause mortality at 12 months but only in patients with ATTRwt-CM presenting with heart failure. Tafamidis was associated with significantly fewer heart failure exacerbations and all-cause mortality (38% reduction) and a higher probability of event-free survival for these endpoints [46].

### Real-world studies evaluating the impact on survival are also lacking

Real-world studies evaluating the impact on survival are also lacking. Hussain et al. [47] conducted a real-world community-based prospective cohort study enrolling 107 patients to evaluate the impact of Tafamidis on survival. Results showed a significant survival benefit associated with Tafamidis (median survival 80.4 vs. 17.2 months, *p* < 0.0001) but in a population predominantly represented by the wtATTR phenotype (94.6%) and median 83.9 years. Thorough confirmation of ATTR was confirmed by biopsy or Tc-99-PYP imaging, 18% of them were presumed to have the wtATTR based on their age. This study agrees with another large prospective cohort (*n*:631) by Bezard et al. [48] which had a great representation of both ATTR subtypes (423 ATTRwt and 225 ATTRv) and a 72% match to the inclusion criteria of the ATTR-ACT trial. Tafamidis was associated with an increase in median major cardiovascular outcome-free survival (*N* = 98): 1565 (1010–2400) days vs. 771 (686–895). Additional real-world analysis has indicated that long-term Tafamidis treatment significantly improved 5-year survival in ATTRwt-CM subsets who were Mayo stage I or II, but not stage III [49].

### Real-world studies evaluating dose-specific efficacy outcomes

One case reported sinus conversion three months after initiation of Tafamidis 80 mg in a 70-year-old male patient with ATTRwt-CM complicated by atrial fibrillation without the need for anti-arrhythmic therapy [50]. This case is important as baseline atrial fibrillation was an independent prognostic factor for all-cause mortality in the ATTR-ACT trial [51].

Sukaina et al. [52] conducted a meta-analysis of three studies including 876 patients randomized to Tafamidis 80 mg only. This included a retrospective study in Japan, ATTR-ACT, and its LTE [10, 18]. Subgroup analysis of cardiovascular mortality with heart failure was also assessed. Results showed that Tafamidis 80 mg was associated with a significant reduction in cardiovascular mortality in both ATTR-CM subtypes. The subgroup analysis revealed a reduction in cardiovascular mortality due to heart failure associated with that 80 mg dose in the Japanese retrospective study [53], but no reduction in the ATTR-ACT trial. This could underline the possibility of heterogeneous responses to Tafamidis in unique populations.

### Real-world studies evaluating efficacy and safety of Tafamidis in diverse populations

Though the most common variant (Val122Ile) in patients of West African descent, there remains a clear discrepancy in access and availability to this drug in developing countries, primarily Africa and the Caribbean [21]. Two observational studies (one retrospective and the other prospective) evaluated the efficacy of Tafamidis in the Japanese population [53, 54]. Even though Nakamura et al.'s study was limited by a small, single-center sample and short follow-up period, there were some noteworthy findings:

Unlike the ATTR-ACT trial which showed an increased rate of rehospitalization in NYHA Classes I and II, this study showed worsening of heart failure symptoms, leading to rehospitalization with longer stays in NYHA Class III patients who were otherwise relatively event-free for one year prior to starting Tafamidis.

Interventricular septal thickness (IVS) worsened with treatment in three patients. Surrogate markers (NT-proBNP) and echocardiographic data remained unchanged from the baseline.

An 83-year-old Korean female with a history of heart failure with preserved ejection fraction (NYHA II) and status post MitraClip intervention presented to the hospital with decreased tolerance during the previous year [55]. The patient was diagnosed with ATTRwt and was started on Tafamidis 20 mg in addition to heart failure therapy. Using magnetocardiography (MCG), the patient was monitored over 4 months. The initial MCG vector was 0.052 before initiation of therapy and after 4 months of therapy, the vector normalized to 0.037. There was an increase in the dose of Tafamidis to 61 mg when the efficacy of the drug seemed to decrease after 27 months of treatment. 18 months later on 61 mg of Tafamidis, the patient reported an improvement in exercise tolerance and an overall improvement in the quality of life during therapy. Restoration of euvolemia and QOL improvement in this patient emphasized the clinical importance of empiric Tafamidis treatment in the early stages of suspecting ATTR-CM [56].

This stabilizing effect of Tafamidis was shown in a case where a 61-year-old patient exhibited signs of decompensated heart failure [57]. After diagnosis with ATTR, she was given diuretic therapy and discharged. She returned six months later with a recurrence of previous symptoms as well as other complications, atrial fibrillation, new complexes on ECG, and pericardial infiltration. Treatment with Tafamidis was initiated empirically and symptoms were resolved after six months she was asymptomatic and reached a stable and fully compensated state [58].

However, there are instances where treatment with Tafamidis is ineffective as in the case of a 72-year-old Korean woman who was diagnosed with Hereditary transthyretin amyloidosis (ATTR) [59]. Tafamidis did not prevent the morphologic, hemodynamic, and histologic dysfunction of amyloid cardiomyopathy. Instrumental investigations carried out before the establishment of the diagnosis included electrocardiography, echocardiography, cardiac magnetic resonance imaging, and endomyocardial biopsy. After 2 years of treatment with Tafamidis, to evaluate the effectiveness of the treatment, the same investigations were carried out with the addition of right-heart catheterization. Signs of worsening amyloid cardiomyopathy were reported which manifested as an increase in the interventricular septal thickness, and myocardial mass. Histologically, there was no significant difference in the TTR amyloid infiltration of the myocardium and loss of cardiomyocytes which supports the notion that Tafamidis inhibits amyloid fibrillogenesis but does not remove infiltrated amyloid from the myocardium. On electrocardiography, a new complete left bundle branch block with QRS prolongation from 109 to 142 ms was revealed in the same patient [60].

It should be noted that the majority of the case reports and clinical trials referenced earlier in this section were funded by the drug manufacturer. Independent case reports may show a slight deviation from the results obtained by case reports obtained by manufacturer-backed reports.

Tafamidis is a strong contender for the treatment of ATTR. Patients have a higher chance of survival and have longer periods of symptom suppression while on Tafamidis. However, there is still some uncertainty when it comes to the reproducibility of clinical trial results in individual patients, thus the need for more widespread use of Tafamidis in clinical practice.

### Challenges and limitations

Clinical trials for rare conditions like ATTR-CM yield small sample sets. This is shown in the ATTR-ACT trial where only 24% of patients represented the ATTR-CMv disease subtype. The parent trial, ATTR-ACT was not a dose-specific assessment as patient-requested dose reductions were allowed. Furthermore, a protocol amendment requiring all patients to switch to the 61 mg tablet (80 mg bioequivalent) resulted in unique durations for each dose. Seven post hoc analyses are reviewed in this article [10, 11, 16, 23–25, 27].

A limitation of post hoc analysis is its rigidity; extracted data from a predetermined sample are used to generate hypotheses based on events that have already occurred. This limits the power of the sub-analysis. One of the studies that evaluated extracted echocardiographic data from the original sample cited a small sample of the ATTRv disease subtype and the inability to infer results over a longer duration as major limitations [23].

A few studies excluded the Tafamidis 20 mg cohort as the 80 mg dose was FDA-approved, causing the lack of generalizability of these results to the 20 mg dose [23, 25, 26]. The analysis did not distinguish between the 20 mg and 80 mg Tafamidis doses; hence, individualized efficacy measures were not ascertained. Patients who discontinued for a reason other than death were assigned as a deterioration, further limiting the power of this study [59].

As with any open-label study, non-blinding bias is high and will affect the integrity of the results.

The pre-specified inclusion criteria of the ATTR-ACT trial automatically excluded elderly and immobile patients, further decreasing the statistical power of this sub-analysis. Low numbers of patients in the > 80 age group joined the LTE study due to expected mortality in this age group, as enrollment occurred 5 years after the parent trial [61]. Patient-reported data for studies assessing long-term impact on health status were used in two studies [24, 27]. There needs to be further studies with clinically objective measures of QOL improvement.

Short follow-up periods in real-world studies cannot support the long-term efficacy of Tafamidis. Meta-analysis comprised mainly retrospective and prospective cohort studies with small samples. Furthermore, the under-representation of hATTR subtypes across the board precluded the generalizability of these findings. Most trials and real-world studies used different doses of Tafamidis preventing consistency in data for a specific dose. Small sample sizes in single-center studies precluding large-scale utilization of Tafamidis have been attributed to its high cost [48, 49]. Two studies [18, 28] used a model-based approach to simulate disease progression; however, this method and the limited pre-specified sample sizes induce variability in survival data.

### Future directions

Despite ATTR-CM's poor prognosis, the current challenge is enhancing awareness of the red flags of the disease. The future of ATTR-CM management is focused on prevention and early diagnosis. Algorithms involving noninvasive, multimodality cardiac imaging, histologic, genetic, and hematological assessments can lead to earlier detection of the disease. Biomarker-based ATTR-CM staging systems have not been thoroughly validated. There is a paucity of data on imaging and biological parameters to support their use in longitudinal follow-up [61]. Therefore, no single staging system is currently in use consistently. Recent research has highlighted the importance of inflammation, oxidative stress, reduced NO availability, thrombosis risk, endothelial dysfunction, and altered vasculature structure, all of which contribute to myocardial remodeling and fibrosis, in addition to amyloid deposition. Unearthing alternative interactions of Tafamidis with the amyloidogenesis pathway and identifying new mediators in its pathophysiology can pave the way forward.

In vivo, DNA editing technologies and anti-TTR monoclonal antibody therapies are on the rise and may prove to be potential competition for Tafamidis in the treatment of ATTR-CM [62]. Most of these drugs are in Phase I or II of development. These therapies have a clear advantage over Tafamidis in that they can get rid of amyloid after it has formed, resulting in potential reversal of disease as opposed to exclusively slowing progression. (Y Ando) Ntla-2001, a DNA editor in Phase 1 development, causes up to 87% TTR knockdown with once annual dosing [63]. The tolerability, safety, pharmacokinetic, and pharmacodynamic profile of two monoclonal antibodies PRX004 and NI006 which promote macrophage-mediated clearance of misfolded forms of TTR are concurrently being evaluated in Phase I trials. (NCT03336580, NCT04360434).

The goal of early diagnosis is to create strategies for earlier detection of ATTR-CM, allowing for earlier intervention with Tafamidis and potentially improving long-term outcomes.

## Conclusion

Over the past decade, clinical trials have endeavored to unravel the efficacy and safety profile of Tafamidis in the treatment of ATTR-CM. Studies have consistently shown that Tafamidis has a significant impact on functional status, cardiovascular-related outcomes, and all-cause mortality with an acceptable safety profile. A greater impact when administered in the early stages of disease is noteworthy. Its high cost, global imbalance of resources and data, and short follow-up periods have precluded the development of larger, real-world trials that could provide more clarity on its long-term efficacy and safety in diverse populations. Several ongoing or planned clinical trials are assessing combined ATTR-CM therapies. Individualized trials focusing on its efficacy in both subtypes are needed. Continuous evaluation of novel disease-specific therapeutics is ongoing.

## Data Availability

Data sharing is not applicable to this article as no datasets were generated or analyzed during the current study.

## Abbreviations

ATTR-CM: Transthyretin amyloid cardiomyopathy
wtATTR: Wild-type Transthyretin
CRISPR: Clusters of Regularly Interspaced Short Palindromic Repeats
TTR: Transthyretin
NYHA: New York Heart Association
QALYs: Quality-adjusted life years
KCCQ-OS: Kansas City Cardiomyopathy Questionnaire Overall Summary
LTE: Long-term extension
NO: Nitric oxide
2D STE: Two-dimensional speckle tracking echocardiography
CMRI: Cardiac magnetic resonance imaging
CS: Compressed sensing
SPECT: Single-photon emission computed tomography
PET: Positron emission tomography
6MWT: 6-minute walk test
SoC: Standard of care
NT-proBNP: N-terminal pro–B-type natriuretic peptide
TEAE: Treatment-emergent adverse event
Tc-99m-PYP: Tc-99 pyrophosphate
99m Tc-DPD: Technetium-99m-labeled 3,3-diphosphono-1,2-propanodicarboxylic acid
LVEF: Left ventricular ejection fraction
LV GLS: LV global longitudinal strain
LTE: Long-term extension
ATTRwt-CM: Wild-type transthyretin amyloid cardiomyopathy
ATTRv-CM: Hereditary or variant transthyretin amyloid cardiomyopathy
